# Effects of Eccentric-Overload vs. Free-Weight High Load Resistance Training on Throwing Velocity in Elite Young Male Handball Players

**DOI:** 10.3390/sports14050172

**Published:** 2026-04-23

**Authors:** Pablo Larrumbide, Gabriel Daza, Víctor Toro-Román, Roger Font, Maria Cadens, Bruno Fernández-Valdés

**Affiliations:** 1 National Institute of Physical Education of Catalonia (INEFC), University of Barcelona (UB), 08038 Barcelona, Spain; larrumbide73@hotmail.com; 2 GRCE Research Group, National Institute of Physical Education of Catalonia (INEFC), University of Barcelona (UB), 08038 Barcelona, Spain; rfont@tecnocampus.cat; 3 Research Group in Technology Applied to High Performance and Health, TecnoCampus, Universitat Pompeu Fabra, Mataró, 08302 Barcelona, Spain; vtoro@tecnocampus.cat (V.T.-R.); bfernandez-valdes@tecnocampus.cat (B.F.-V.); 4 Research Group into Human Movement, National Institute of Physical Education of Catalonia (INEFC), University of Lleida (UdL), 25192 Lleida, Spain; mcadens@gencat.cat

**Keywords:** flywheel, one repetition maximum, team sports, performance

## Abstract

Throwing velocity is a key performance factor in handball and may be enhanced through strength training. The aim of the present study was to quantify improvements in throwing velocity in handball players and to compare the effects of a free-weight strength training programme (FW; *n* = 14; 18.07 ± 1.27 years; 86.19 ± 9.67 kg; 1.85 ± 0.08 m) and a flywheel-based eccentric overload training programme (FLYW; *n* = 13; 17.77 ± 1.17 years; 85.5 ± 8.38 kg; 1.85 ± 0.06 m). A total of 27 elite male youth handball players (*n* = 27; 17.93 ± 1.21 years; 85.86 ± 8.90 kg; 1.85 ± 0.07 m) participated in the study. Participants were allocated to groups using a stratified randomisation approach based on team and playing position. Of these, 14 performed the FW training program and 13 completed the FLYW training protocol. The FW group performed 3 sets of 6 repetitions at 80% of 1RM, with 3 min of rest between sets, using the exercises half squats, bench presses and pullovers. The FLYW training group trained with flywheel devices, executing 3 sets of 6 repetitions using four inertial loads, performing each repetition at maximal intended velocity, with 3 min of rest between sets, using the exercises unilateral press, overhead elbow extension, and trunk rotation. Both groups trained twice per week for 8 weeks, in combination with regular handball-specific training. Pre- and post-intervention assessments included the indirect estimation of one-repetition maximum (1RM) in the half squats, bench presses, and pullovers, as well as throwing velocity. The FW group showed significant improvements in all variables (bench press, half squat, pullover, and throwing velocity; all *p* < 0.05). In contrast, the FLYW group showed significant improvements only in half squats (*p* = 0.034) and throwing velocity (*p* = 0.008). An 8-week strength training program using free weights and flywheel methods improved throwing velocity in elite youth handball players; however, neither method demonstrates clear superiority when throwing velocity is the primary outcome.

## 1. Introduction

Handball is a team sport played between two opponents on a 40 × 20 m court [1,2]. It is considered an intermittent sport involving frequent high-intensity actions and physical contact [3]. During matches, players combine general movements such as walking, running, or jumping with sport-specific actions such as passing, throwing, or blocking [4].

Throwing is a fundamental skill in handball. Throwing velocity is a key factor that influences its success [5]. The faster the ball is thrown, the less time defenders and/or the goalkeeper have to react. The main factors influencing throwing velocity include the sequencing of body segments, the technique used, and the strength and power of both upper and lower limbs [2,4]. Based on this, throwing velocity may be enhanced through strength training [5,6]. However, there is some disagreement regarding which type of resistance training is most effective for improving throwing velocity.

The review by Petruzela et al. [7] suggests that training aimed at developing throwing velocity in handball should include general resistance exercises such as the bench press, performed at moderate to high intensities. Specifically, training at intensities above 80% of one-repetition maximum (1RM) may effectively improve throwing velocity [8,9]. This response to high-intensity loads may be attributed to neural adaptations associated with strength training [7].

Other studies have implemented exercises with greater specificity, showing dynamic correspondence with the throwing gesture [10]. Dynamic correspondence, as described by Verkhoshansky & Siff [11], refers to how much a training task directly improves an athlete’s sport performance because it matches the specific skills of the sport. This relationship is commonly known as the “transfer effect”. For instance, Andersen et al. [12] improved throwing velocity in young female handball players by integrating resistance band exercises. Similarly, Raeder et al. [13] observed improvements in throwing velocity in amateur female players through medicine ball throwing exercises.

Although eccentric overload training using flywheel devices has been shown to enhance strength and muscle mass [14,15], there is limited evidence regarding its effects on throwing performance in handball. Saez de Villareal et al. [16] reported improvements in throwing performance in the Spanish second division male handball players following a flywheel training program (FLYW) that included both upper and lower body exercises with flywheel devices. Likewise, Maroto-Izquierdo et al. [17] found throwing performance improvements in professional handball players from the men’s first division in Spain following a shoulder-specific FLYW intervention.

It is well established that FLYW training elicits greater muscle activation during the eccentric phase compared to traditional resistance exercises [18]. This type of training provides a unique stimulus that closely resembles plyometric loading in the stretch-shortening cycle (SSC) [19,20]. Given that the throwing action involves a sequence of eccentric followed by concentric contractions, it may benefit from such stimuli [9]. Furthermore, flywheel devices allow movement to be executed freely across all three planes, enabling the design of more complex and specific exercises with greater dynamic correspondence [21,22].

To our knowledge, despite growing evidence supporting both training modalities independently, no studies have been found in the literature directly comparing eccentric overload training using flywheel devices with traditional free-weight (FW) strength training in terms of improving throwing performance in handball players. Therefore, the aim of the present study was to evaluate the effects of an FW training program and an FLYW training program on throwing velocity in handball players. We hypothesized that FLYW training would lead to greater improvements in throwing velocity compared to traditional FW exercises. This hypothesis was based on three key mechanisms: (1) enhanced eccentric muscle activation during flywheel training [15], (2) greater SSC involvement similar to the throwing motion [9], and (3) the biomechanical possibility of designing exercises with higher dynamic correspondence to the throwing pattern, including multi-planar rotational movements [22].

## 2. Materials and Methods

### 2.1. Participants

An a priori sample size calculation was performed using G*Power (version 3.1, Heinrich-Heine-Universität Düsseldorf, Germany) for a repeated-measures ANOVA (within–between interaction). Assuming an effect size of f = 0.30, α = 0.05, and power (1 − β) = 0.80, the required total sample size was estimated at 24 participants. Therefore, the sample size included in this study (*n* = 27) was considered sufficient.

A total of 27 high-level male youth handball players participated in the study. They belonged to the under-18 and second team of a top 3 club in Spain’s first division handball league, with several having experience with the youth national team. Participants were recruited through convenience sampling, representing a highly trained and specialized population [23]. The participants’ characteristics are presented in Table 1.

Among the total number of participants, 14 players followed the FW strength training programme, while the remaining 13 participated in the FLYW strength training programme. The study employed a quasi-experimental design, with group allocation determined by team and playing position using a stratified randomization approach. No significant differences were observed between groups at baseline for age (*p* = 0.526), height (*p* = 0.961), body mass (*p* = 0.846), or training experience (*p* = 0.519).

During the season, the players completed 3 weekly strength training sessions (90 min long), 4 weekly specific handball training sessions and 1 match day corresponding to the youth league of the first national division.

All participants or their parents/legal guardians provided informed consent by signing a formal consent form. The study was conducted in accordance with current ethical standards, as outlined in the Declaration of Helsinki [24] and was approved by TecnoCampus (Pompeu Fabra University) Institutional Ethics Committee (code 6/2024).

The criteria to participate in the study were as follows: (i) at minimum of 6 years of experience playing handball; (ii) experience of at least 3 years in strength training; (iii) complete at least 75% of the sessions of the strength training program; (iv) no long-term injury (at least 2 months) in the last 3 months prior to the study; (v) maintain a similar lifestyle (physical activity and nutrition) throughout the research period.

### 2.2. Study Design

A quasi-experimental design was used to determine the effects of strength training interventions (FW or FLYW) on throwing velocity. A 2-month (8-week) strength training program was implemented, with sessions held twice a week (16 sessions in total). Participants performed the resistance training programs in addition to the same handball-specific sessions for both groups.

Two weeks prior to starting the resistance training programs, participants completed 2 familiarization sessions, which consisted of 3 sets of 6 repetitions of each exercise from the FLYW training using the flywheel device with 4 inertias, and 3 sets of 6 repetitions with FW exercises at an RIR (repetitions in reserve) of 4. After the familiarization period, participants performed 16 sessions (2 sessions per week for 8 weeks) of either FW training or FLYW training.

**Figure 1:**
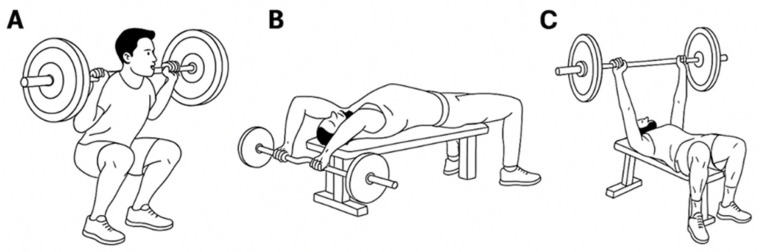
Free-Weight Training. (**A**): Half squat; (**B**): Pull Over; (**C**): Bench Press.

The FW training consisted of bench press, half squat, and pull over exercises (Figure 1). Three sets of 6 repetitions at 80% of 1RM were performed for each exercise, with 3 min of rest between sets. Participants were instructed to perform all repetitions at maximal intended velocity during the concentric phase.

**Figure 2:**
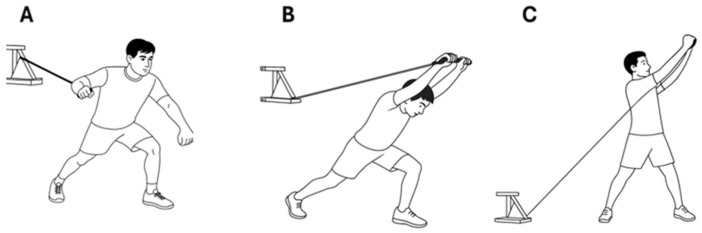
Flywheel Training. (**A**): Unilateral Press; (**B**): Overhead Elbow Extension; (**C**): Trunk Rotation.

The flywheel training consisted of a unilateral press, an overhead elbow extension and a trunk rotation (Figure 2). Three sets of 6 repetitions were performed for each exercise with 4 inertias, executing each repetition at the maximum possible speed, with 3 min of rest between sets.

The unilateral press was performed as a single-arm push with the contralateral leg in a forward stance, using the pulley positioned at mid-height. The pushing action was accompanied by slight hip and knee flexion during the eccentric phase, followed by hip and knee extension during the concentric phase (Figure 2A).

The overhead elbow extension was carried out as a bilateral elbow extension performed above the head, with one leg in a forward stance, using the pulley positioned at a high setting. Players coordinated the bilateral overhead elbow extension with an alternating change of the forward leg (Figure 2B).

The trunk rotation exercise was executed with the pulley set at a low position, involving a diagonal rotational movement of the trunk. As in the unilateral press, trunk rotation was accompanied by hip and knee flexion during the eccentric phase, and hip and knee extension during the concentric phase (Figure 2C).

The order of the exercises was varied in each session to avoid prioritizing any particular exercise. During each session, strength and conditioning coaches supervised the exercises and encouraged participants to move the load as quickly as possible. The sessions were conducted prior to handball-specific training for each group.

### 2.3. Assessment

Four days prior to the commencement of the resistance training programs and four days after their completion, the 1RM for bench press, half squat, and pull over, as well as throwing velocity, were tested.

- One repetition maximum

Before and after the strength training programs, the 1RM for each exercise was assessed to prescribe the training load during the program. The evaluation involved the indirect calculation of the 1RM, following Brzycki’s guidelines [25]. The 1RM was calculated using Brzycki’s original formula, as shown below:

1RM = load (kg)/(1.0278 + 0.0278 × reps)

The Brzycki formula is a valid method for evaluating 1RM in exercises such as bench press and squat [26,27,28].

The warm-up and loading protocols were identical on both testing days. Participants began with an initial 5-min light aerobic warm-up on a stationary bike at a perceived exertion of 9–11 on the Borg 6–20 scale (light effort) [29], followed by dynamic stretching of the main muscle groups involved in the exercises. Subsequently, participants performed the specific evaluated movement with a light load. The total warm-up duration ranged between 10 and 15 min.

After the warm up, participants performed approach sets to 1RM. Before starting the approach phase, the athletes were asked about their best 1RM mark for each exercise to determine their level and prescribe the approach series correctly.

The approach protocol consisted of an initial series of 12 repetitions without added weight (only bar), trying to mobilize as much force as possible in the concentric contraction, stopping for a second at the end of the range of motion and controlling the movement in the eccentric phase.

After the initial warm-up sets, a series of 2–3 approximation repetitions was performed, with the load being increased freely based on the athlete’s capability, ensuring a 60-s recovery period between sets. Participants were instructed to complete the concentric phase of the movement as quickly as possible. If the execution speed noticeably decreased, visually, repetitions were continued until failure, which was always less than 5 maximum repetitions (5RM) [25]. Once voluntary failure was reached by the participant, the load and the maximum number of valid repetitions were recorded. Throughout the testing procedure, players were encouraged to exert maximum effort.

- **Bench press**

During the bench press, participants assumed a supine position on a weight bench, ensuring five points of contact (head, glutes, and both shoulders on the bench, and both feet flat on the floor). Participants lifted the bar from the rack and extended their arms. Participants lowered the bar until it touched the chest, then pressed it upward until the arms were fully extended. They returned the bar to the starting position, maintaining tight abdominal muscles and a stable body position without bouncing or arching their back.

- **Half squat**

During the performance of the half squat the participant began in an upright position, with his knees and hips fully extended, and the bar on his back in a position directly under the spine of the scapula, just below the posterior deltoid. All participants had their feet flat on the floor, and once the bar was lowered, no change in posture was allowed until the bar had to be replaced in the holder at the end of the test. Participants maintained an upright position. The bar was grasped firmly with both hands and was also supported on the shoulders. The knees were bent to 90 degrees and the subject then regained the upright position, with the legs fully extended.

- **Pull over**

The pull over exercise was performed in a supine position on a weight bench, ensuring five points of contact (head, glutes, and both shoulders on the bench, and both feet flat on the floor). Participants lifted the bar from the rack and extended their arms. The eccentric action took the weight over and behind the individual’s head, with the elbow fully extended. At the end of the backward movement, when the upper limbs were approximately parallel to the ground and the elbows were slightly flexed, subjects pushed the barbell to bring it back to the starting position, keeping their abdominal muscles well contracted and their body stable without bouncing or arching their back.

- **Ball throwing velocity**

Throwing velocity was evaluated using a standing throw with a run-up with a standard handball ball (mass 480 g, circumference 58 cm). For the standing throw players initiated with a bounce from midfield (20 m from the goal) and took three approach steps to throw at maximum velocity into a 1-m diameter ring at the center of the goal without stepping over the 9-m line [30]. Each player completed three throws with a 2-min interval between throws. The throw with the highest velocity was recorded using a radar positioned 2 m behind the goal at a height of 1 m. Players were permitted to apply resin to their hands, and participants were instructed to use their preferred technique.

All three throws were measured using a radar (Stalker Sport, Applied Concepts Inc, Richardson, TX, USA), positioned 2 m behind the goal at a height of 1 m. The researchers ensured that the throwing test adhered to the established rules. Subjects were promptly informed of their performance to motivate them.

### 2.4. Statistical Analysis

A two-way repeated-measures analysis of variance (ANOVA) was performed to evaluate the effects of the training interventions on the dependent variables. The model included one within-subject factor (Time: pre vs. post) and one between-subject factor (Group: FW vs. FLYW).

The interaction effect (Group × Time) was used to determine whether the changes over time differed between training programs. When significant interactions were observed, post hoc analyses with Bonferroni correction were conducted.

Effect sizes were reported as partial eta squared (η^2^p) and interpreted as small (0.01), medium (0.06), and large (0.14). Statistical significance was set at *p* ≤ 0.05.

**Figure 3:**
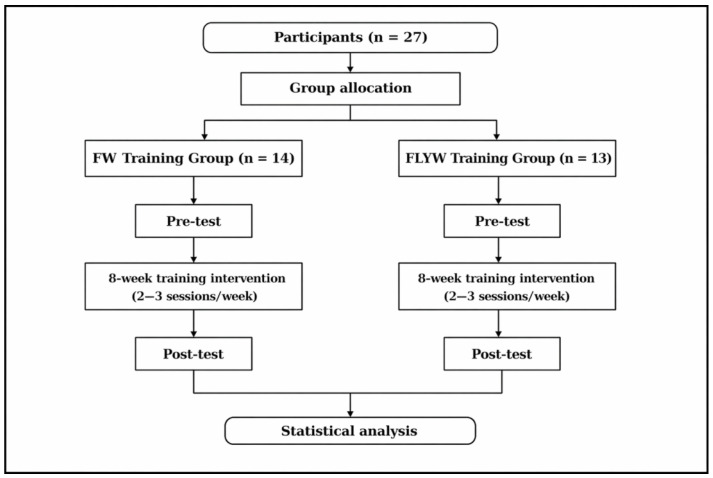
Flow diagram of the study design.

The study design is shown in a flow diagram in Figure 3.

## 3. Results

A two-way repeated-measures ANOVA (Group × Time) was performed to evaluate the effects of the training interventions on performance variables. (Table 2).

**Figure 4:**
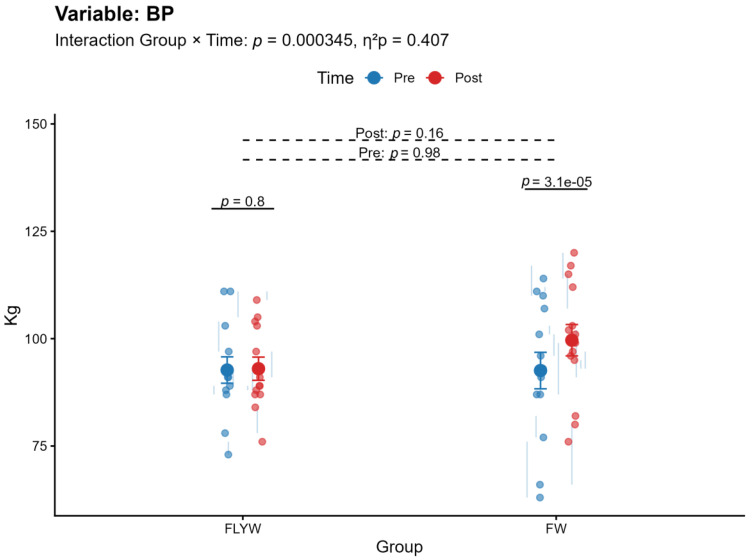
Pre- and post-intervention changes in bench press (BP) in the flywheel training group (FLYW) and the free-weight training group (FW).

**Figure 5:**
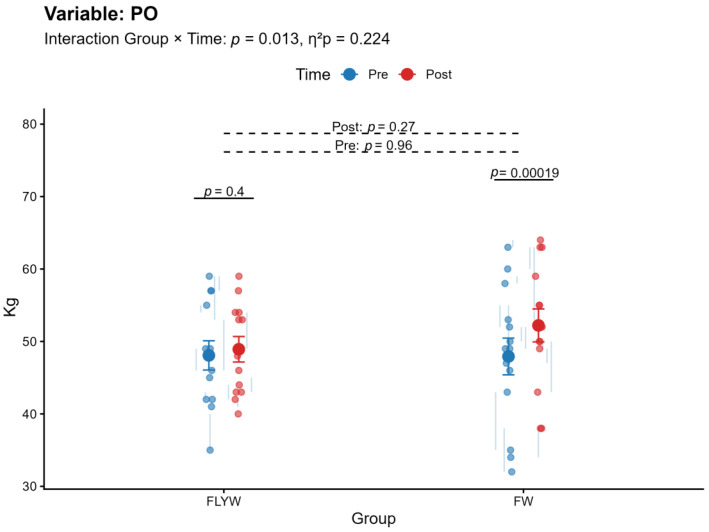
Pre- and post-intervention changes in pull over (PO) in the flywheel training group (FLYW) and the free-weight training group (FW).

**Figure 6:**
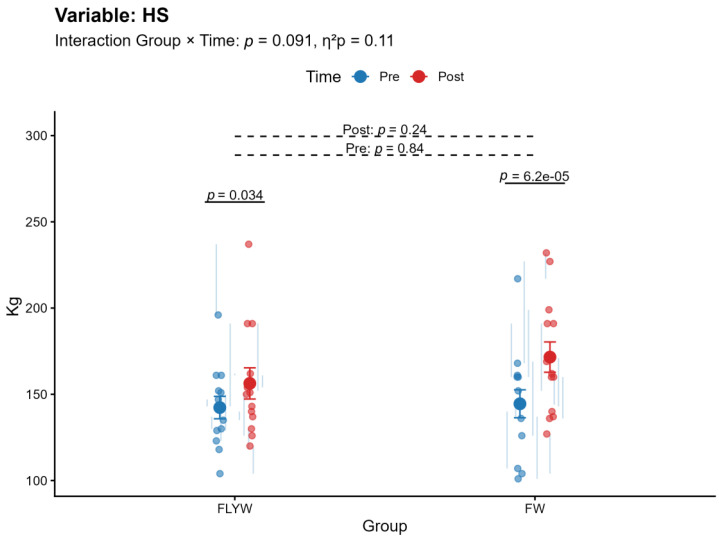
Pre- and post-intervention changes in half squat (HS) in the flywheel training group (FLYW) and the free-weight training group (FW).

**Figure 7:**
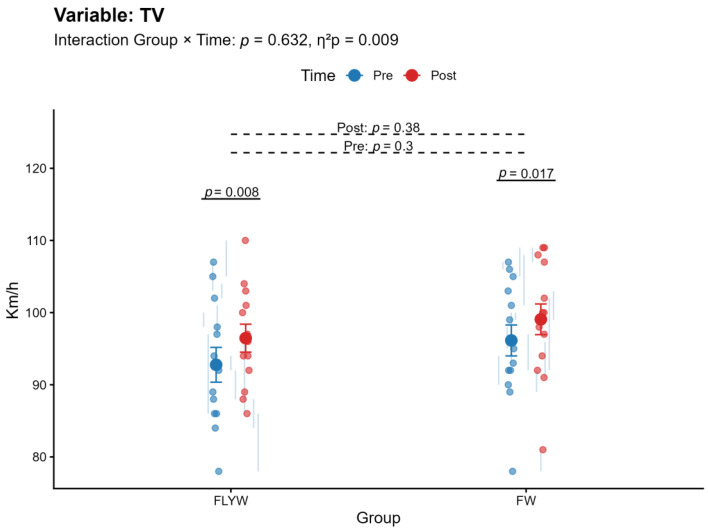
Pre- and post-intervention changes in throwing velocity (TV) in the flywheel training group (FLYW) and the free-weight training group (FW).

A significant Group × Time interaction was observed for bench press (BP) (*p* < 0.001, η^2^p = 0.407; Table 2; Figure 4) and pull-over (PO) (*p* = 0.013, η^2^p = 0.224; Table 2; Figure 5), indicating that the magnitude of change differed between the FW and FLYW groups. No significant interaction was found for half squat (HS) (*p* = 0.091, η^2^p = 0.110; Table 2; Figure 6) or throwing speed (TS) (*p* = 0.632, η^2^p = 0.009; Table 2; Figure 7).

Regarding within-group changes, the FW group showed significant improvements in all variables (BP, HS, PO, and TS; all *p* < 0.05). In contrast, the FLYW group showed significant improvements in HS (*p* = 0.034) and TS (*p* = 0.008), but not in BP (*p* = 0.80) or PO (*p* = 0.40) (Table 3).

Between-group comparisons revealed no significant differences between FW and FLYW at baseline (all *p* > 0.05), confirming group equivalence prior to the intervention. Similarly, no significant differences were observed between groups at post-intervention (all *p* > 0.05) (Table 4).

Overall, although both training programs improved performance, the FW group demonstrated greater improvements in upper-body strength variables (BP and PO), as reflected by the significant interaction effects. (Figure 4 and Figure 5).

## 4. Discussion

The main aim of this study was to compare the effects of flywheel (FLYW) and free-weight (FW) resistance training on throwing velocity in elite youth handball players. The main finding was that both training modalities significantly improved throwing velocity; however, no significant Group × Time interaction was observed for this variable, indicating that the magnitude of improvement was comparable between the two training approaches.

In line with these findings, significant Group × Time interactions were observed for the bench press and pullover, suggesting that the FW training program induced greater improvements in upper-body maximal strength compared to FLYW training. Nevertheless, both methods were effective and may lead to different neuromuscular adaptations.

Improvements were observed in the FW group, consistent with the findings of Petruzela et al. [7], who suggested that training with loads around 80% of 1RM is optimal for enhancing maximal strength and throwing performance. Numerous studies have examined the effects of strength training programs on 1RM and throwing performance [8,9,31,32,33], demonstrating that strength gains from heavy-load training are influenced by both morphological and neural factors [34]. Increases in muscle cross-sectional area are considered a key determinant of maximal force production [35], while neural adaptations may occur through increased motoneuron output, leading to greater motor unit recruitment and firing rates, ultimately enhancing force production [36,37,38].

Highly significant improvements in 1RM were observed across all exercises in the FW training program. These findings are consistent with previous research reporting substantial improvements following high-load strength training interventions. Marques and González-Badillo [33] reported a 28% increase in bench press 1RM in professional handball players after 12 weeks of training at intensities between 70–95% of 1RM. Similarly, Hermassi et al. [9] observed increases of over 50% and 20% in the pullover, and 10% and 5% in the bench press, following 10 weeks of training with high and moderate loads, respectively. Sabido et al. [39] reported improvements of 9% in bench press using known loads and 10.1% using unknown loads after a 4-week intervention at intensities of 30–50–70% of 1RM in junior handball players.

Increases in maximal strength should theoretically provide players with an advantage in sustaining the high levels of force required during throwing actions [8,9]. In this regard, Hermassi et al. [9] reported a 43% improvement in standing throw velocity following high-load training with pullover and bench press exercises over 10 weeks in first-division players. Similarly, Hermassi et al. [32] observed significant improvements using comparable exercises at intensities of 80–95% of 1RM. Marques and González-Badillo [33] reported increases of 4% after 6 weeks and 6% after 12 weeks, while Sabido et al. [39] found a 4.7% improvement after 4 weeks using unknown loads in the bench press.

Interestingly, the FLYW group improved throwing velocity despite showing minimal changes in upper-body maximal strength. These improvements were achieved without meaningful increases in 1RM values for the bench press or pullover, with significant changes observed only in the half squat, likely due to the involvement of the lower body in all FLYW exercises. It should also be considered that the FW group may have achieved greater improvements in 1RM due to the closer similarity between the training intervention and the testing tasks, potentially enhancing familiarisation effects.

The improvements in throwing velocity are consistent with previous studies. Saez de Villarreal et al. [16] reported a 7.85% increase in standing throw velocity following a flywheel-based intervention similar to that used in the present study, while Maroto-Izquierdo et al. [17] observed a 4.1% improvement in throwing velocity at 7 m in professional handball players after an eccentric overload training program targeting the shoulder.

Several mechanisms may explain these findings. Greater muscle activation during the eccentric phase, compared to FW exercises [18], together with increased involvement of the stretch–shortening cycle (SSC), may have contributed to the observed improvements in throwing performance. Similarly, Hermassi et al. [9] also reported significant enhancements in throwing performance following training programs that emphasized SSC utilization with high loads. In addition, the design of the FLYW tasks likely played a key role, as they demonstrated a high degree of dynamic correspondence with the throwing action, incorporating high-velocity rotations of the pelvis, trunk and shoulders under overload conditions. Similar findings have been reported in interventions using medicine balls, which also exhibit high movement specificity and have been shown to improve throwing performance in handball [13]. In baseball, pulley-based training interventions involving multiplanar, high-velocity movements have also yielded improvements in pitching performance [40]. Furthermore, the selected exercises involved substantial activation of the core musculature, which is essential for the efficient transfer of energy from proximal to distal segments during throwing actions [41].

Overall, these findings support the idea that both traditional resistance training and more specific training modalities can improve throwing performance in handball players, although neither approach appears to be clearly superior when throwing velocity is considered the primary outcome.

From a practical perspective, FW training appears to be more effective for developing maximal strength, whereas FLYW training may provide a more specific stimulus related to sport performance. Therefore, combining both approaches could represent an effective strategy to optimize both strength development and performance outcomes.

Finally, several limitations should be acknowledged. The absence of a control group and the relatively small sample size may limit the generalizability of the findings. In addition, differences in exercise specificity between training programs may have influenced the results. Future research should explore combined training strategies and examine their effects across different phases of the competitive season.

## 5. Conclusions

In summary, an 8-week strength training intervention combined with sport-specific handball training, incorporating high-load FLYW or FW protocols, improves standing throw velocity in elite youth handball players.

The results revealed different patterns of improvement in maximal strength between the two training approaches. Those who trained with free weights increased their performance in all one-repetition maximum tests, whereas the flywheel group improved only in the half squat. This difference suggests that each method produces a particular type of adaptation, highlighting the need to choose exercises according to the specific goal of the training program—whether it is to develop general strength or to enhance throwing performance.

Taken together, these observations indicate that eccentric overload training with a flywheel can be considered a practical option, or even a useful complement, to conventional high-load strength training when the aim is to increase throwing velocity in handball players. Nevertheless, more research is required to understand how both methods can be most effectively integrated throughout the different stages of an annual training cycle.

## Figures and Tables

**Table 1:** Participant characteristics.

	Free-Weight Training	Flywheel Training
***n***	14	13
**Age (years)**	18.07 ± 1.27	17.77 ± 1.17
**Weight (kg)**	86.19 ± 9.67	85.5 ± 8.38
**Height (m)**	1.85 ± 0.08	1.85 ± 0.06
**Experience (years)**	8.71 ± 1.59	9.08 ± 1.26

**Table 2:** ANOVA (Group × Time).

Variable	Effect	*p*-Value	η^2^p
BP	Group × Time	<0.001	0.407
HS	Group × Time	0.091	0.110
PO	Group × Time	0.013	0.224
TS	Group × Time	0.632	0.009

**Table 3:** Between groups.

Variable	Group	*p*-Value	Interpretation
BP	FLYW	0.80	No change
BP	FW	<0.001	Significant increase
HS	FLYW	0.034	Significant increase
HS	FW	<0.001	Significant increase
PO	FLYW	0.40	No change
PO	FW	<0.001	Significant increase
TS	FLYW	0.008	Significant increase
TS	FW	0.017	Significant increase

**Table 4:** Pre vs. Post (within-group).

Variable	Time	*p*-Value	Interpretation
BP	Pre	0.98	No difference
BP	Post	0.16	No difference
HS	Pre	0.84	No difference
HS	Post	0.24	No difference
PO	Pre	0.96	No difference
PO	Post	0.27	No difference
TS	Pre	0.30	No difference
TS	Post	0.38	No difference

## Data Availability

The data are not publicly available due to privacy and ethical restrictions.

## Abbreviations

The following abbreviations are used in this manuscript:

FLYW	Flywheel training program
FW	Free weight
RM	Repetition maximum
SSC	Stretch-shortening Cycle
RIR	Repetition in reserve
